# Deciphering the mechanisms of action of progesterone in breast cancer

**DOI:** 10.18632/oncotarget.28455

**Published:** 2023-07-01

**Authors:** Gaurav Chakravorty, Suhail Ahmad, Mukul S. Godbole, Sudeep Gupta, Rajendra A. Badwe, Amit Dutt

**Affiliations:** ^1^Integrated Cancer Genomics Laboratory, Advanced Centre for Treatment, Research and Education in Cancer, Kharghar, Navi Mumbai 410210, Maharashtra, India; ^2^Homi Bhabha National Institute, Training School Complex, Mumbai 400094, Maharashtra, India; ^3^Department of Biosciences and Technology, Faculty of Sciences and Health Sciences, Dr. Vishwanath Karad MIT World Peace University, Pune 411038, Maharashtra, India; ^4^Department of Medical Oncology, Tata Memorial Hospital, Tata Memorial Centre, Navi Mumbai 410210, Maharashtra, India; ^5^Department of Surgical Oncology, Tata Memorial Hospital, Tata Memorial Centre, Navi Mumbai 410210, Maharashtra, India

**Keywords:** breast cancer, progesterone, *DSCAM-AS1*, endocrine therapy

## Abstract

A practice-changing, randomized, controlled clinical study established that preoperative hydroxyprogesterone administration improves disease-free and overall survival in patients with node-positive breast cancer. This research perspective summarizes evidences from our studies that preoperative hydroxyprogesterone administration may improve disease-free and overall survival in patients with node-positive breast cancer by modulating cellular stress response and negative regulation of inflammation. Non-coding RNAs, particularly *DSCAM-AS1*, play a regulatory role in this process, along with the upregulation of the kinase gene *SGK1* and activation of the *SGK1/AP-1/NDRG1* axis. Progesterone-induced modification of the progesterone receptor and estrogen receptor genomic binding pattern is also involved in orchestrating estrogen signaling in breast cancer, preventing cell migration and invasion, and improving patient outcomes. We also highlight the role of progesterone in endocrine therapy resistance, which could lead to novel treatment options for patients with hormone receptor-positive breast cancer and for those who develop resistance to traditional endocrine therapies.

## INTRODUCTION

Breast cancer is one of the most common cancer types worldwide, accounting for approximately 25% of all female cancers. According to the World Health Organization, an estimated 685,000 women succumbed to breast cancer in 2020, making it the fifth leading cause of cancer-related deaths overall [1]. Additionally, about 1 in 8 women worldwide are prone to develop breast cancer in their lifetime, and the mortality rate for patients with this disease varies depending on various factors, such as age, reproductive factors, disease stage, and access to appropriate treatment [2]. Surgical resection of the primary tumor is often the first-line of treatment for patients with operable or early-stage breast cancer [3]. Depending on multiple factors, including expression of steroid hormone receptors in tumors, surgery may be followed by adjuvant therapies, such as chemotherapy, radiation therapy, or hormonal therapy to reduce the risk of recurrence [4]. Intriguingly, clinical evidence suggests that women with breast cancer who receive surgical resection during the luteal phase of menstrual cycle have better survival outcomes than those who undergo surgery during other stages [5]. This therapeutic benefit may be attributable to the preventive impact of elevated progesterone levels during the luteal phase of the menstrual cycle in breast cancer [6]. To test the hypothesis, in a landmark phase III randomized clinical trial on patients with operable breast cancer, a single dose of hydroxyprogesterone was administered before surgery to mimic the luteal (progestogenic) phase; the analyses demonstrated a significantly increased duration of disease-free survival in patients with node-positive breast cancer, suggesting a protective effect of progesterone in breast cancer [7]. Of the several hypothesis-generating results from this trial, the survival benefit conferred to patients with breast cancer raised an important question about how progesterone improves the survival outcome? This research perspective is aimed to highlight the multipronged roles of progesterone in breast cancer that underlie the associated clinical response. We also present our opinion on the role of progesterone in overcoming endocrine resistance in the patients, especially by countering the genomic effects of overexpression and mutation of *ESR1* and its targets.

### Progesterone regulates *SGK-1/AP-1/NDRG1* genomic axis and stress response in breast cancer cells

Preoperative hormonal treatments, as opposed to neoadjuvant chemotherapy, are simpler to administer, easier to monitor, less expensive, and cause less toxicity [8]. Therefore, a better understanding of the targets of hormonal therapy could be of tremendous potential in monitoring the response in human cancers. We investigated the molecular basis of action of hydroxyprogesterone, a biosimilar form of natural progesterone, that patients with operable breast cancer received as a single injection prior to surgery. In the study, we performed whole transcriptome sequencing (RNA-Seq) of primary breast tumor samples collected before and after hydroxyprogesterone exposure, as well as a control group of patients who underwent only surgery. The results suggested 207 genes to be significantly altered between the progesterone-exposed and -unexposed groups; of which, 142 genes were upregulated post-surgery in progesterone-exposed patients. A pathway enrichment analysis identified genes that respond to progesterone to be associated with cellular stress, nonsense-mediated decay of proteins, and negative regulation of inflammatory response to be the most deregulated pathways [9]. Specifically, the study revealed that preoperative hydroxyprogesterone manifests its effect in patients with breast cancer by downregulating genes involved in inflammatory response and production of TNF that are known to induce proliferative, invasive, and malignant behavior of breast cancer cells. The study also identified upregulation of a tumor metastasis suppressor gene, *N-Myc downstream regulated gene 1*, *NDRG1*, along with increased expression of the AP-1 network genes, suggesting that preoperative progesterone intervention may modulate the effect of surgical stress on breast cancer by altering the expression of several protein-coding genes, thereby improving patient survival [9]. Additionally, a significant deregulation of the central node ubiquitin gene *UBC*, in the study, in response to progesterone, further underlined the downstream stress-regulating activities modulated by progesterone in breast cancer cells, potentially independent of the progesterone receptor (PR) [9]. Overall, these findings indicate that pre-operative hydroxyprogesterone may act to curb cellular stress responses and potentially mediate the pro-survival effects through a negative regulation of inflammation.

**Figure 1:**
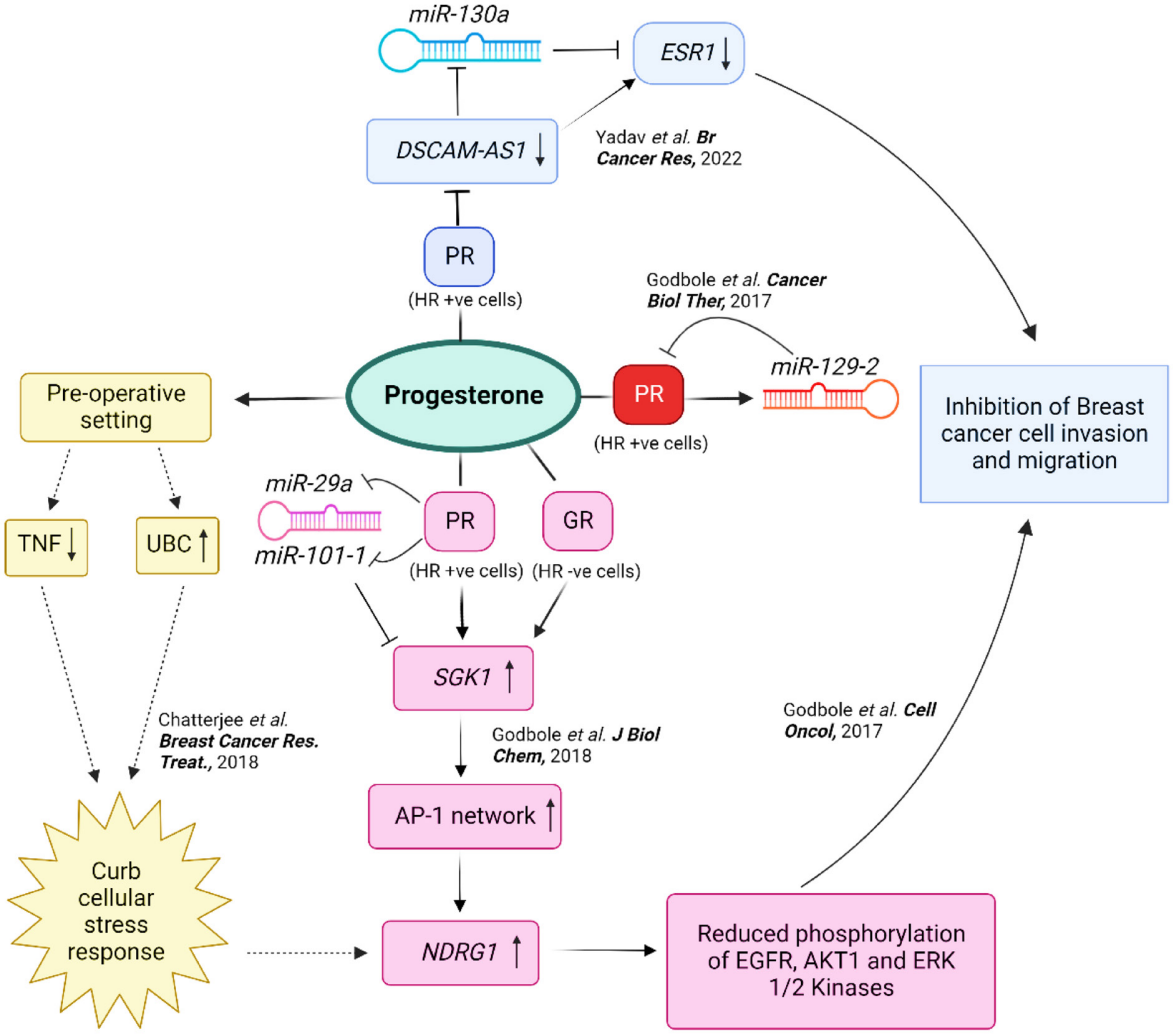
An integrated representation of the multifaceted effects of progesterone in breast cancer. A schematic representation to describe the molecular mechanisms by which progesterone acts to curb cellular stress response and promote cell survival in breast cancer cells. Whole transcriptomic studies (represented by dotted lines) suggest that progesterone downregulates genes involved in the inflammatory response and *TNF* production while upregulating the central node ubiquitin gene *UBC* and *N-myc downregulated gene 1*, *NDRG1*, in primary breast tumors. Thus, the effects of progesterone are mediated through a negative regulation of inflammation. Biochemical and genetic studies (represented by solid lines) suggest that progesterone treatment of breast cancer cells *in vitro* increases the expression of a *serum- and glucocorticoid-regulated kinase 1*, *SGK1*, which upregulates *NDRG1* via the *AP-1* network genes, independent of the *PR* status of the cells. Progesterone also suppresses the expression of *miR-29a* and *miR-101-1* that target the 3′-UTR of *SGK1*, reflecting a dual-regulatory mode of expression of *SGK1* in breast cancer. The increased expression of *NDRG1* reduces the phosphorylation of kinases, and thus, suppress cell invasion and migration of the cells, providing a mechanism for the previous study. Progesterone-mediated upregulation of *miR-129-2* also decreases the expression of *PR* in breast cancer. Additionally, progesterone treatment downregulates the expression of *DSCAM-AS1* in breast cancer cells that sponges *miR-130a* targeting the 3′-UTR of *ESR1*, and suppresses the migration and invasion of *PR*-positive breast cancer cells. This model provides a molecular basis for the clinical findings of preoperative progesterone intervention in breast cancer. Abbreviation: HR: Hormone Receptor.

In a separate study, we conducted functional analysis of the components found to be significantly altered after performing an integrated genomic profiling of a panel of PR-positive and PR-negative breast cancer cell lines treated with progesterone to investigate the molecular action of progesterone in breast cancer cells [10]. The study led to an intricate convergence model indicating a dual-phase regulation downstream to progesterone to regulate the expression of its direct transcriptional target *serum- and glucocorticoid-regulated kinase 1*, *SGK1*, and downregulation of *miR-29a* and *miR-101-1* targeting *SGK1* in breast cancer cells. We showed that the upregulation of *SGK1* led to the activation of *NDRG1*, mediated by AP-1 network genes to inactivate AKT1, ERK1/2, and EGFR kinases, and thus, inhibit breast cancer cell invasion and migration [10]. The increased expression of *NDRG1* upon progesterone treatment in breast cancer tissue samples and cell lines confirms the progestogenic- and stress-mediated genomic regulation of *NDRG1* [9, 10]. This mechanism provided a better understanding of how progesterone reduces breast cancer cell invasion and migration independent of PR status [11]. Moreover, we observed an enriched binding of PR, estrogen receptor (ER), and p300 at the *SGK1* genomic locus to enhance the expression of *SGK1*; this finding corroborates with previous analyses that progesterone alters the ER genomic activity in breast cancer [12]. The study also established that glucocorticoid receptor (GR) mediates the progesterone effect in PR-negative breast cancer cells [10]. Interestingly, we observed a widespread expression of membrane PRs (mPR, PGRMC1) in both PR-positive and PR-negative breast cancer cells, indicating that additional mechanisms could mediate progesterone functions [10]. Taken together, the study suggests that preoperative exposure to progesterone could benefit patients with operable breast cancer by modulating the surgical stress response, observed in our previous study. This modulation may be mediated by the upregulation of the kinase gene *SGK1*, which activates the *AP-1*/*NDRG1* axis in both progesterone receptor (PR)-positive and -negative breast cancer cells. The activation of the *SGK1/AP-1/NDRG1* axis is significant because it is involved in regulating cellular stress responses. *NDRG1* is a stress response gene [13] involved in regulating the response to surgical stress in breast cancer cells. Our study suggests that increased expression of *NDRG1* by preoperative progesterone exposure may help to mitigate the negative effects of surgical stress on breast cancer cells. Similarly, the kinase gene *SGK1* has also been implicated in regulating cellular stress responses. It is involved in regulating the response to oxidative stress and in promoting cell survival under stress conditions [14]. As this effect was consistently observed in both PR-positive and -negative breast cancer cells, it suggests that the benefits of preoperative progesterone exposure may not be limited to patients with PR-positive cancers. This finding is of clinical importance as a significant proportion of patients have PR-negative cancers and may fail to respond to adjuvant or neoadjuvant endocrine therapies that target the PR pathway. Additionally, the study highlights targeting specific microRNAs and their target genes as a therapeutic strategy for improving breast cancer outcomes (Figure 1).

### Progesterone modulates the *DSCAM-AS1/miR-130a/ESR1* genomic axis in breast cancer

Several studies have identified the regulatory functions of noncoding microRNAs and long noncoding RNAs (lncRNAs) in breast cancer progression [15, 16]. Our group has previously identified progesterone-responsive microRNAs that regulate expression of *PR* and *SGK1* in breast cancer [10, 17]. While the regulation of lncRNAs by estrogen has been extensively studied [15, 18], the role of progesterone in the regulation of lncRNAs in breast cancer cells is unclear. Only a few studies have identified progesterone-responsive lncRNAs in lung and endometrial cancer cells [19, 20]. LncRNAs function as microRNA sponges or competitive endogenous RNAs (ceRNA) that regulate functions of microRNAs in cancer cells [21]. For instance, several lncRNAs, such as *HULC* [22], *HOTAIR* [16], *HOXD-AS1* [23], and *MIAT* [24] show ceRNA activity. Moreover, lncRNAs can function to enhance the expression of steroid hormone receptors such as *ESR1* [25]. These complex interactions between noncoding RNAs and steroid hormones and receptors highlight the need for further research to fully understand the mechanisms underlying breast cancer progression and develop new therapeutic strategies. However, lncRNAs regulated by progesterone in breast cancer remain largely unknown.

In a recent study, we described the regulatory role of noncoding RNAs in response to progesterone to mediate the cellular changes [26]. In brief, the whole transcriptome sequencing dataset of hydroxyprogesterone-exposed breast primary tumors and control samples were re-analyzed to identify differentially expressed noncoding RNAs followed by functional characterization of the deregulated lncRNA. We identified downregulation of a lncRNA, *Down syndrome cell adhesion molecule antisense RNA 1*, *DSCAM-AS1*, upon progesterone treatment in breast cancer cells. The genetic silencing of *DSCAM-AS1* mimicked progesterone treatment as it reduced breast cancer cell invasion and migration. Interestingly, *DSCAM-AS1* is known to be upregulated in response to estrogen and promotes ER-positive breast cancer progression [15]. Specifically, our study revealed that progesterone suppresses the expression of *DSCAM-AS1* by altering ER binding at the *DSCAM-AS1* genomic locus, consistent with previous reports [10, 12]. *DSCAM-AS1* functions as a microRNA sponge for *miR-130a*, which is similarly sponged by *DSCAM-AS1*, targets the 3′-UTR of *ESR1* that is a crucial regulator of breast cancer progression and metastasis [27, 28]. The miRNA sponge activity of *DSCAM-AS1* has been earlier reported for *miR-101* [29], *miR-186* [30], and *miR-136* [31] in osteosarcoma and endometrial cancer. These results highlight a novel interplay between *DSCAM-AS1* and *ESR1* via *miR-130a*, downstream to progesterone. Thus, progesterone opposes the estrogen–ER signaling at the *DSCAM-AS-1–ESR1* genomic axis via two synergistic modes: it reduces the expression of *DSCAM-AS1* and elevates the expression of *miR-130a*, which interacts with both *DSCAM-AS1* and 3′-UTR of *ESR1* in breast cancer cells. Reduction in the levels of *ESR1* via the *DSCAM-AS1/miR-130a* axis also correlates with a decrease in the cancerous properties of cells, impeding breast cancer cell invasion and migration, similar to progesterone treatment [11, 26]. Additionally, in patients with breast cancer, high expression of *miR-130a* or low expression of *DSCAM-AS1* correlates with better survival outcomes, similar to progesterone treatment [7, 26]. *DSCAM-AS1* and *miR-130a* may thus serve as biomarkers for predicting survival outcomes in patients with PR-positive breast cancer (Figure 1). In addition, the integrated analyses highlighted pronounced cellular effects of the *DSCAM-AS1/miR-130a/ESR1* genomic axis in PR-positive breast cancer cells, with similar significant effects observed on survival of patients with ER-positive breast cancer. Therefore, the expression patterns of *DSCAM-AS1* and *miR-130a* may aid physicians in better categorizing patients with the luminal A/B cancer subtype to provide them with the proper therapeutic care and assistance in extending their life outcomes.

### Progestogen orchestrates estrogen signaling and influences outcomes of endocrine resistance in breast cancer

The presence of PR in breast cancer is a well-established prognostic factor, with patients with PR-positive cancers tending to have a better prognosis than those with PR-negative cancers [32]. However, recent studies have suggested that progesterone and PR may also play a role in endocrine therapy resistance in breast cancer [12, 33]. Studies suggest that PR can induce the expression of genes involved in cell survival and proliferation, such as Bcl-2 and cyclin D1, which may contribute to endocrine therapy resistance [34, 35]. In addition, progesterone and mPRs have been shown to interact with other oncogenic pathways, such as the PI3K/AKT/mTOR pathway, which are also implicated in endocrine therapy resistance [36]. Interestingly, lncRNAs have been shown to participate in mediating resistance to endocrine therapy in breast cancer [37–39]. Of note, *DSCAM-AS1* has been implicated in conferring resistance to tamoxifen in breast cancer [15]. We show that knockdown of *DSCAM-AS1* leads to decreased *ESR1* transcript levels, while overexpression of *DSCAM-AS1* leads to increased *ESR1* expression [26]. The regulation of *DSCAM-AS1* expression by ER and PR in breast cancer cells in response to progesterone suggests a potential feed-forward mechanism between *DSCAM-AS1* and *ESR1* in breast cancer. This finding is particularly relevant given that overexpression of *ESR1* has been shown to cause estrogen-independent growth in preclinical breast cancer models, which is associated with endocrine therapy resistance [15]. Moreover, the overexpression of *DSCAM-AS1* in tamoxifen-resistant cell line models, which also overexpress *ESR1*, highlights the potential role of *DSCAM-AS1* in endocrine therapy resistance in breast cancer [15, 26, 40]. These findings provide new insights into the mechanisms underlying the effects of progesterone on breast cancer progression and resistance to endocrine therapy. They also have implications for the development of novel therapeutic strategies targeting PR/ER signaling in breast cancer. Finally, the progesterone-induced downregulation of *PR*, a target of *ESR1*, further supports the hormonal interplay between progesterone and estrogen signaling pathways in breast cancer [17].

The interplay between progesterone and estrogen signaling in breast cancer is complex, and recent research suggests that the use of combinatorial endocrine therapies requires careful consideration to avoid pro-tumorigenic outcomes. While progesterone has shown promise in modulating the efficacy of endocrine therapies such as tamoxifen, fulvestrant, and giredestrant against *ESR1*-mutant breast cancer, the outcomes of such treatments have also been pro-tumorigenic [41]. In contrast, preclinical models of breast cancer have shown an additive antitumorigenic response upon treatment with progesterone and endocrine therapy [12]. This suggests that further understanding of the role of progesterone in endocrine therapy resistance may lead to the development of new therapeutic strategies for patients with steroid hormone receptor-positive breast cancer who fail to respond to standard endocrine therapies. Therefore, it is crucial to carefully consider the use of combinatorial endocrine therapies in breast cancer treatment and to continue investigating the complex interplay between progesterone and estrogen signaling in breast cancer. Ultimately, this will help researchers develop more effective and personalized treatment strategies for patients with breast cancer. The WinPro clinical trial (NCT03906669) is a significant step in investigating the potential benefits of adding synthetic progesterone (prometrium) to endocrine therapy in postmenopausal patients with early breast cancer [42]. This trial may provide valuable insights into the effects of progesterone on breast cancer cells and its interaction with endocrine therapies. By studying the mechanisms underlying endocrine therapy resistance, researchers can gain a better understanding of how to develop more effective treatments for this disease. The results of the WinPro trial and other similar studies may help clarify the role of progesterone in breast cancer treatment and provide a basis for developing new therapeutic strategies for patients with this disease. Taken together, the investigation of the complex interplay between progesterone and endocrine therapies in breast cancer is crucial for improving patient outcomes and developing more personalized treatment approaches. The ongoing research in this area holds great promise for the future of breast cancer treatment.

## CONCLUSIONS AND FUTURE PERSPECTIVES

The preoperative hydroxyprogesterone administration improves disease-free and overall survival in patients with node-positive breast cancer. The mechanism behind this improvement is thought to be related to the modulation of cellular stress response and the negative regulation of inflammation through the upregulation of the kinase gene *SGK1*. Activation of the *SGK1/AP-1/NDRG1* axis, which is involved in regulating cellular stress responses, may play a significant role in this process. Our study also highlights the regulatory role of non-coding RNAs in response to progesterone and their potential contribution to cellular changes that affect breast cancer prognosis. We have identified the downregulation of a lncRNA called *DSCAM-AS1* in breast cancer cells treated with progesterone. Further analyses showed that the *DSCAM-AS1/miR-130a/ESR1* genomic axis had a more pronounced effect on the prognosis of patients with ER-positive breast cancer. Progesterone-induced modification of the PR and ER genomic binding pattern orchestrates estrogen signaling in breast cancer, preventing cell migration and invasion, and improving the prognosis of patients with breast cancer.

Our findings highlight the complex genomic and molecular mechanisms involved in the response to progesterone in breast cancer and the potential for non-coding RNAs to play a regulatory role in this process. The mechanisms underlying the observed effects of progesterone on breast cancer outcomes are unclear. However, progesterone may modulate the expression of genes involved in tumorigenesis and metastasis, as well as affect the expression and genomic activity of ER and other factors that play a critical role in breast cancer development and progression. Further studies are needed to elucidate the precise mechanisms by which progesterone influences breast cancer outcomes. This information may help in the development of novel treatment and diagnostic strategies to overcome this disease.

Overall, while preoperative hydroxyprogesterone administration appears to be a promising strategy for improving the prognosis of patients with node-positive breast cancer, the investigation of the mechanism of actions of progesterone in breast cancer remains an important area of research that holds great promise for improving patient outcomes.
